# Vitamin E for the Prevention of Chemotherapy-Induced Peripheral Neuropathy: A meta-Analysis

**DOI:** 10.3389/fphar.2021.684550

**Published:** 2021-05-13

**Authors:** Jie Chen, Haili Shan, Wenjun Yang, Jiali Zhang, Haibin Dai, Ziqi Ye

**Affiliations:** ^1^ Department of Pharmacy, The Second Affiliated Hospital, Zhejiang University School of Medicine, Hangzhou, China; ^2^ Department of Clinical Pharmacy, The First Affiliated Hospital, Zhejiang University School of Medicine, Hangzhou, China

**Keywords:** vitamin E, CIPN, incidence of all-grade peripheral neuropathy, total neuropathy scores, meta-analysis

## Abstract

**Background:** Vitamin E has been increasingly used to prevent chemotherapy-induced peripheral neuropathy (CIPN) in recent years. However, it is still unclear whether vitamin E can effectively prevent CIPN.

**Methods:** We searched all clinical studies in the Embase, Cochrane Library, Clinicaltrials.gov, and PubMed databases from inception to December 2020. We performed a meta-analysis of 9 randomized controlled trials (RCTs) with 486 patients that compared the vitamin E group with the control group. Outcomes of the study were incidence of all-grade CIPN, incidence of severe CIPN, and the total neuropathy scores (TNS). Random effect models were used to make the meta-analysis results more cautious.

**Results:** Notably, vitamin E significantly reduced the incidence of all-grade CIPN (overall risk ratio (RR) = 0.55, 95% CI: 0.36, 0.85, I^2^ = 77.3%, *p* = 0.007), and TNS (overall standard mean difference (SMD) = −0.64, 95% CI: **−**1.03, −0.25, I^2^ = 42.7%, *p* = 0.001). However, the results of the subgroup analysis, which included only double-blind RCTs, suggested that vitamin E did not significantly reduce the incidence of all-grade CIPN (overall RR = 0.52, 95% CI: 0.07, 4.06, I^2^ = 77.5%, *p* = 0.531). Moreover, there was no significant difference in the incidence of severe CIPN between these two arms (*p* = 0.440).

**Conclusion:** The results of our meta-analysis suggests that vitamin E has a beneficial effect on the incidence and symptoms of CIPN. However, routine prophylactic use of vitamin E is still not recommended. Moreover, more high-quality double-blind RCTs are needed to further validate the effects of vitamin E in prevention of CIPN.

## Introduction

Chemotherapy-induced peripheral neuropathy (CIPN) is a dose-limiting toxicity caused by chemotherapy drugs, such as cisplatin, oxaliplatin, bortezomib, vinca alkaloids, and taxanes (Selvy et al., 2021). The incidence of CIPN ranges from 19 to 85%, and it depends mainly on the chemotherapy regimen and the dosage (Fallon, 2013; Brami et al., 2016). Other important factors affecting the incidence of CIPN are the different methods of screening and diagnosis. The diagnosis of CIPN is based mainly on the patient’s description of symptoms and the physician’s examinations. The most frequently used assessment tools are questionnaires, which completed by the physicians or the patients whose different perceptions of the severity of CIPN may lead to a biased diagnosis (Loprinzi et al., 2020). A meta-analysis showed that the incidence of CIPN was 68.1% at 1 month after chemotherapy, 60.0% at 3 months, and 30.0% at 6 months. Notably, the high incidence of CIPN in cancer patients can significantly affect the patients’ long-term quality of life (QoL) (Seretny et al., 2014).

CIPN, symptomized by pain, paresthesia or burning sensation, is mainly a sensory neuropathy. In contrast, motor neuropathy, generally symptomized by muscle weakness, shows lower incidence (Samuels and Ben-Arye, 2020). The mechanism is usually due to direct toxic effects (e.g., platinum-based drugs) or disruption of microtubules (e.g., taxanes, and vinca alkaloids) (Carlson and Ocean, 2011). Although the etiology of CIPN is not well understood, an evidence shows that free radicals, referred to as reactive oxygen species (ROS), can result in some CIPN induced by certain chemotherapeutic agents (Waseem et al., 2018). Mitochondrial dysfunction and oxidative stress are the main mechanisms of platinum-based drug-induced CIPN. High intracellular concentrations of ROS can lead to the destruction of enzymes, proteins and lipids, further leading to structural changes in peripheral nerves. ROS also induces apoptosis of neuronal cells via the mitochondrial pathway. It can also activate the caspase pathway and dysregulate calcium homeostasis, a process that leads to the loss of dorsal root ganglion (DRG) cells (Zajaczkowska et al., 2019).

**FIGURE 1:**
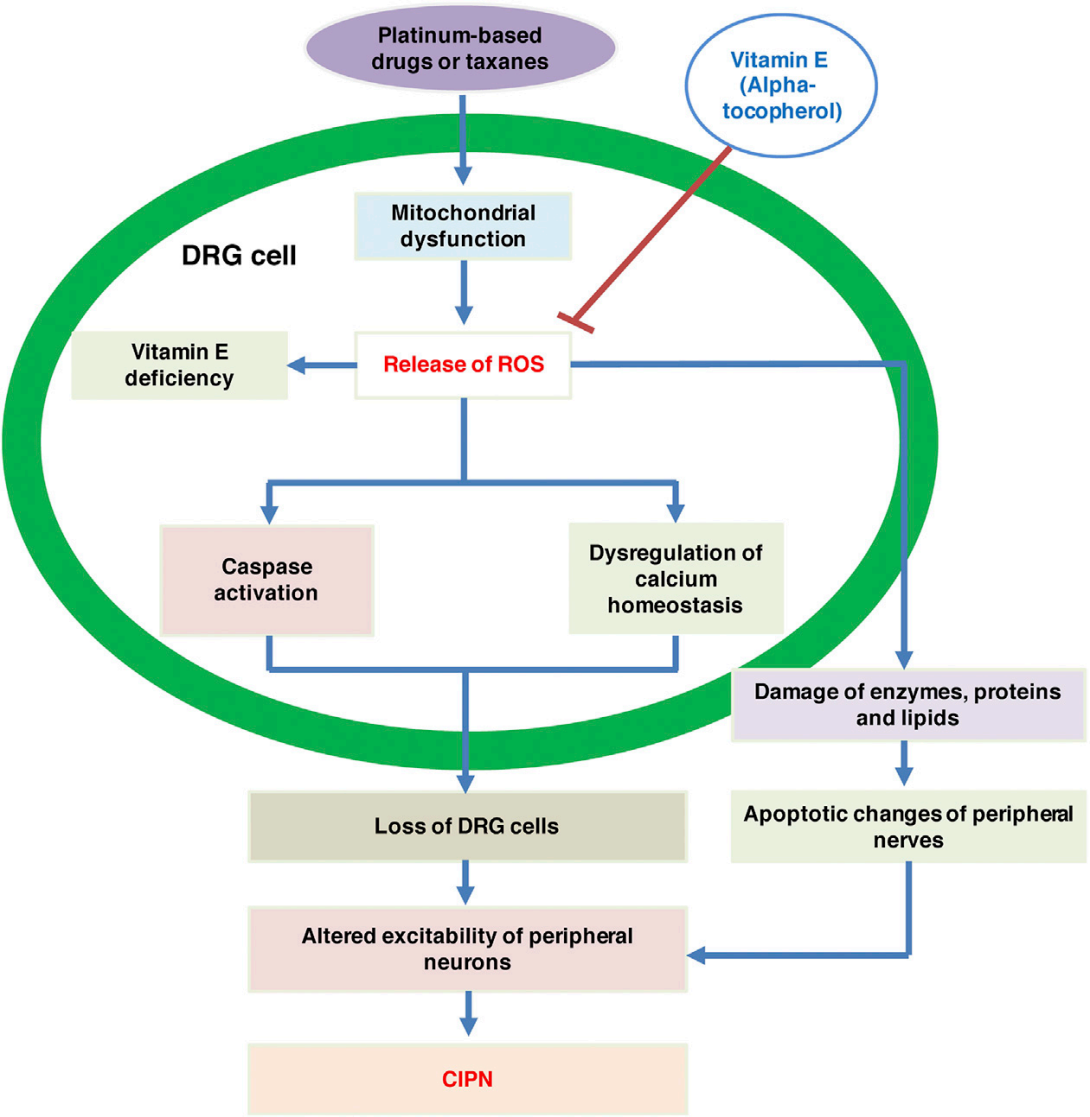
The possible mechanisms of vitamin E in CIPN. CIPN, chemotherapy-induced peripheral neuropathy; DRG, dorsal root ganglion; ROS, reactive oxygen species.

Animal studies have suggested that antioxidant supplementation can provide protection against various chemotherapy toxicities associated with oxidative stress (Rybak et al., 2000). Vitamin E, one of the most widely studied antioxidants, contains tocopherols and tocotrienols, and the most biologically active component of vitamin E is alpha-tocopherol (Malik et al., 2019). The results of one clinical study demonstrated that plasma levels of vitamin E decreased significantly in cancer patients who developed severe neuropathy after cisplatin treatment. In another group of patients, plasma levels of vitamin E also decreased significantly after 2 and 4 cycles of cisplatin treatment (Bove et al., 2001). Studies have shown that vitamin E deficiency affects the central and peripheral nervous systems and might lead to peripheral neuropathy. Therefore, it was thought that vitamin E supplementation could prevent the development of CIPN or alleviate existing CIPN-related symptoms (Samuels and Ben-Arye, 2020). Additionally, studies have confirmed that vitamin E can be used safely and effectively in CIPN caused by taxanes and cisplatin (Anoushirvani et al., 2018). In summary, the possible mechanisms of vitamin E in CIPN are shown in Figure 1.

To date, the prevention of CIPN remains a challenge. Neither the American Society of Clinical Oncology (ASCO) guidelines nor the European Society for Medical Oncology (ESMO) guidelines have recommended any drugs that can be used to prevent CIPN (Jordan et al., 2020; Loprinzi et al., 2020). The conclusions of the ASCO guidelines were based primarily on a meta-analysis that included six RCTs and concluded that prophylactic administration of vitamin E did not reduce the risk of CIPN (RR = 0.55, 95% CI: 0.29,1.05, *p* = 0.07). This conclusion was supported by another small clinical study (Loprinzi et al., 2020). Similarly, the conclusions of the ESMO guidelines were based on another meta-analysis that included three RCTs and concluded that vitamin E only had a slight subjective benefit for CIPN (Jordan et al., 2020). However, a meta-analysis which included 5 randomized controlled trials (RCTs) showed that vitamin E supplementation (300–600 mg/d) was significantly effective in preventing CIPN (Eum et al., 2013). Although there is growing evidence that vitamin E can be used to prevent CIPN, this conclusion remains controversial. Since more data have been available, we conducted this meta-analysis to more comprehensively assess whether vitamin E was effective in preventing CIPN.

## Materials and Methods

### Statement

All clinical studies included were published and had ethical approval, and we did not collect or utilize raw data from these studies; therefore, no further ethical approval was required. We conducted this meta-analysis based on the requirements of the Priority Reporting Items for Systematic Evaluation and Meta-Analysis (PRISMA) (the PRISMA checklist of this meta-analysis were listed in Supplementary Table S1) (Page et al., 2021).

### Literature Search and Study Selection

We searched all clinical studies that compared the vitamin E group and control group in the Embase, Cochrane Library, Clinicaltrials.gov (http://clinicaltrials.gov/), and PubMed databases from inception to December 2020. We exclusively included clinical trials that were published in English. The search terms included: “Vitamin E,” “Tocopherol,” “Neuropathy,” “Peripheral neuropathy,” “Chemotherapy-induced peripheral neuropathy,” and “CIPN.” The references of the articles included in this study were also searched for a more comprehensive screening of the literature.

The inclusion criteria for clinical studies in this meta-analysis included full texts of RCTs comparing the vitamin E group and control group with regards to CIPN. Outcomes of the study were incidence of all-grade CIPN, incidence of severe CIPN, and total neuropathy scores (TNS). Included studies should report at least one outcome, and appropriate assessments were required at the end of the intervention or after randomization. To avoid duplication, if several clinical studies recruited patients during the same period or at the same study center, we only chose the studies with the largest sample size or the most relevant results. Either open-label or double-blind RCTs were included in this meta-analysis. Exclusion criteria included case reports, reviews, nonclinical studies, editorials, and abstracts.

Two reviewers independently screened the titles of the clinical studies and checked the full text or abstracts for confirmation of eligibility. When disagreement occurred, two reviewers discussed the issue first, and if consensus could still not be reached, a third reviewer was involved.

### Data Extraction and Quality Assessment

Two reviewers independently extracted the following data from the eligible clinical studies: study name, country, study type, sample size, age, chemotherapy regimens, dosage of vitamin E, experimental and control group interventions, diagnostic method of CIPN, and outcomes. The TNS was used to assess the neurotoxic conditions of the patients based on their neuropathological signs and symptoms and electrophysiological changes. Studies included in this meta-analysis used TNS (mild: 1–4, moderate: 5–8, and severe: >8), modified peripheral neuropathy (PNP) scores (mild: 1–11, moderate: 12–23, and severe: >24), or Common Terminology Criteria for Adverse Events (CTCAE), version 3.0 (mild: 1, moderate: 2, severe: 2+) to grade the severity of CIPN. As mentioned previously, the incidence of all-grade CIPN (including mild, moderate, and severe CIPN), incidence of severe CIPN, and TNS were the outcomes to be pooled.

We assessed the methodological quality of RCTs according to the five dimensions of the Jadad criteria: description of randomization, appropriate method for randomization, description of double-blind, appropriate method for double-blinding, and description of withdrawals and dropouts (Janda and Swiston, 2012). We scored the quality of each included RCT study and categorized it as either a high-quality study or a low-quality study. High-quality studies were defined as Jadad scores ≥3, whereas low-quality studies were defined as Jadad scores ≤2.

### Statistical Analysis

We used STATA version 12.0 software to perform the statistical analysis. Outcomes were assessed by calculating the standardized mean difference (SMD) or risk ratio (RR), along with the respective 95% confidence intervals (CIs). We used the Cochrane Q test to assess heterogeneity among the included clinical studies, and the I^2^ statistic was applied to check the magnitude of heterogeneity. If I^2^ was greater than 50%, the results were considered to be significantly heterogeneous. To make the results more rigorous, we used a random effects model to calculate the pooled effects and their respective 95% CIs. When there was a significant heterogeneity in the results, subgroup analysis was performed to find the sources of heterogeneity, and sensitivity analysis was also used to assess the robustness of the calculated results. A funnel plot was used to qualitatively analyze the publication bias in the included studies. Meanwhile, the Begg adjusted rank correlation test was used to quantitatively assess the publication bias (Hu et al., 2019). A *p* value of less than 0.05 was considered to be statistically significant.

## Results

### Study Selection and Characteristics of Studies

**FIGURE 2:**
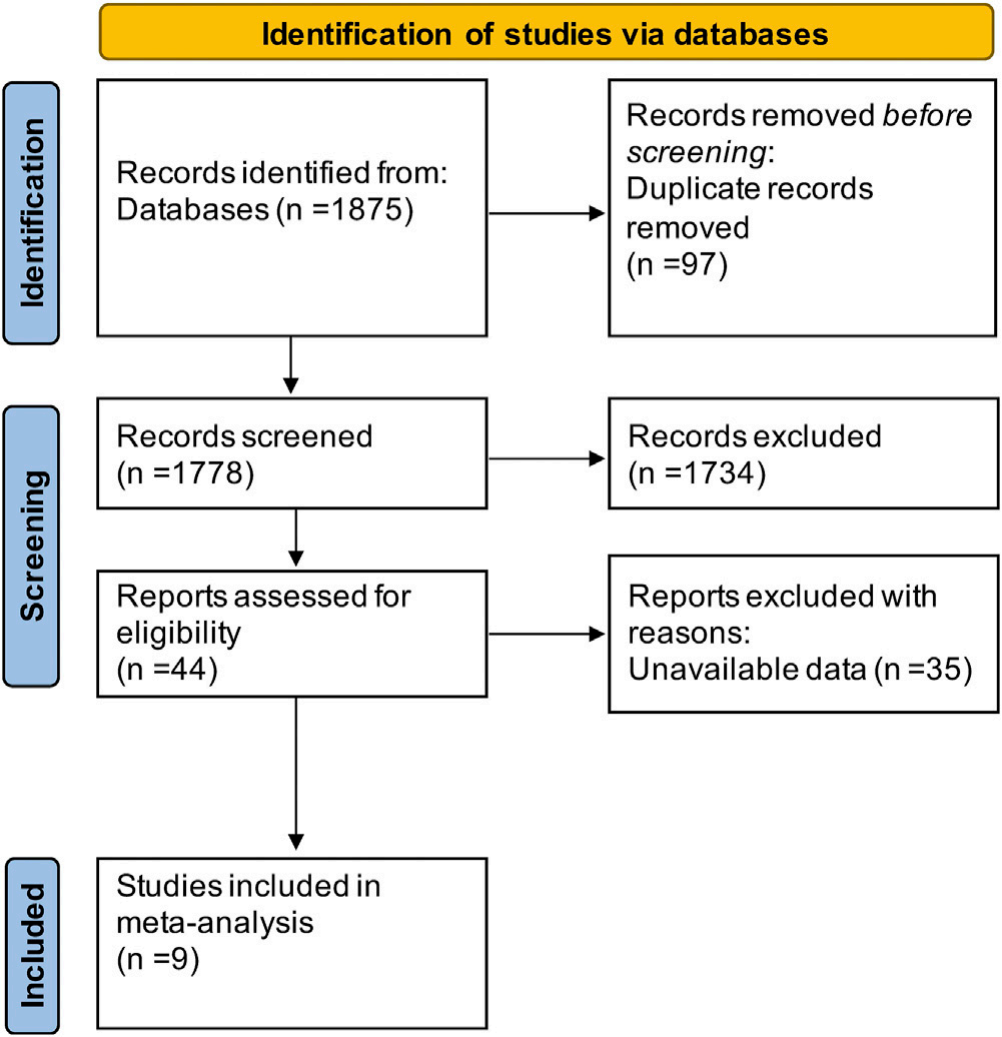
Flow chart of studies screening.

The literature search resulted in a total of 1,875 articles (shown in Figure 2), of which 97 duplicates were removed and 1,734 articles were excluded because they were animal studies, abstracts, reviews, or articles unrelated to the content of this study. Aditionally, we excluded 35 articles that could not extract at least one outcome by screening the full text. Finally, 9 RCTs (Pace et al., 2003; Argyriou et al., 2005; Argyriou et al., 2006a; Argyriou et al., 2006b; Pace et al., 2010; Kottschade et al., 2011; Afonseca et al., 2013; Salehi and Roayaei, 2015; Anoushirvani et al., 2018) including 486 patients were enrolled in this study. All articles were published in English, and Table 1 shows the detailed characteristics of the included studies.

**TABLE 1:** Characteristics of included studies.

References	Studies	Country	Trials type	Sample size		Age (Mean ± SD);		Chemotherapy	Dosage of VE	Outcomes	Diagnostic method of CIPN
				VE	Con	VE	Con				
Anoushirvani et al. (2018)	Pace et al. (2003)	Italy	RCT	13	14	58.0 ± 8.2	57.0 ± 11.7	Cisplatin	300 mg/d	IPN, TNS	NA, CP, NE
Pace et al. (2003)	Argyriou et al. (2005)	Greece	RCT	16	15	55.8 ± 12.6	57.5 ± 11.4	Cisplatin, Paclitaxel	600 mg/d	IPN	NA, CP, NE
Argyriou et al. (2005)	Argyriou et al. (2006b)	Greece	RCT	18	19	56.8 ± 8.3	57.2 ± 11.5	Paclitaxel	600 mg/d	IPN, TNS	NA, CP, NE
Argyriou et al. (2006a)	Argyriou et al. (2006a)	Greece	RCT	14	16	53.6 ± 14.8	59.3 ± 12.8	Cisplatin	600 mg/d	IPN, TNS, AE	NA, CP, NE
Argyriou et al. (2006b)	Pace et al. (2010)	Italy	RCT	17	24	58.0 ± 11.0	58.5 ± 10.7	Cisplatin	400 mg/d	IPN, TNS	NA, CP, NE
Pace et al. (2010)	Kottschade et al. (2011)	USA	RCT	96	93	/	/	Taxanes, Cisplatin, carboplatin, Oxaliplatin, or combination	600 mg/d	IPN	NA, CP
Kottschade et al. (2011)	Afonseca et al. (2013)	Brazil	RCT	18	16	56.0 ± 12.0	57.0 ± 7.9	Oxaliplatin	400 mg/d	IPN, AE	NA, CP
Afonseca et al. (2013)	Salehi et al. (2015)	Iran	RCT	32	33	56.0 ± 14.3	58.9 ± 13.6	Oxaliplatin	400 mg/d	IPN, TNS	NA, CP
Salehi and Roayaei (2015)	Anoushirvani et al. (2018)	Iran	RCT	21	21	50.9 ± 10.4	52.2 ± 10.1	Paclitaxel	600 mg/d	IPN	NA, CP, NE

RCT, randomized controlled trial; VE, vitamin E; Con, control; IPN, incidence of peripheral neuropathy; TNS, total neuropathy scores; AE, adverse events; NA, neurologic assessment; CP, clinical presentation of CIPN; NE, Neurophysiological examination.

### Methodological Quality of Individual Studies

Eighty-nine percent (8/9) of the included clinical studies were of high quality (Jadad scores ≥3), and only one article was evaluated as low quality (Jadad scores = 2), mainly because it did not elaborate on the method used for randomization. The methodological quality of individual studies included in this meta-analysis is detailed in Table 2.

**TABLE 2:** Quality assessment of included studies.

Studies	Description of randomization	Appropriate method for randomization	Description of double-blind	Appropriate method for double-blinding	Description of withdrawals and dropouts	Jadad scores
Pace 2003	1	0	0	0	1	2
Argyriou 2005	1	1	0	0	1	3
Argyriou 2006	1	1	0	0	1	3
Argyriou 2006	1	1	0	0	1	3
Pace 2010	1	1	1	1	1	5
Kottschade 2011	1	1	1	1	1	5
Afonseca 2013	1	1	1	1	1	5
Salehi 2015	1	1	0	0	1	3
Anoushirvani 2018	1	1	0	0	1	3

### Assessment of Incidence of CIPN

The assessment/diagnosis of CIPN in the studies included in this meta-analysis was described as follows: all patients were examined neurologically by a neurologist prior to chemotherapy to document sensory/motor nerve abnormalities and deep tendon reflexes. The loss of Achilles reflexes was usually reported as the first manifestation of CIPN. A follow-up neurological examination was performed by the same neurologist after the cessation of chemotherapy. In addition, neuropathic signs and symptoms were assessed using specially designed scales such as the Neurological Symptom Score (NSS), Neurological Disability Score (NDS), and Hughes’ Functional Grading Scale (FGS). Well-tolerated neurophysiological examinations, such as nerve conduction studies (NCS) and electromyography (EMG), were also performed in some of the included studies for the diagnosis of CIPN (listed in Table 1).

**FIGURE 3:**
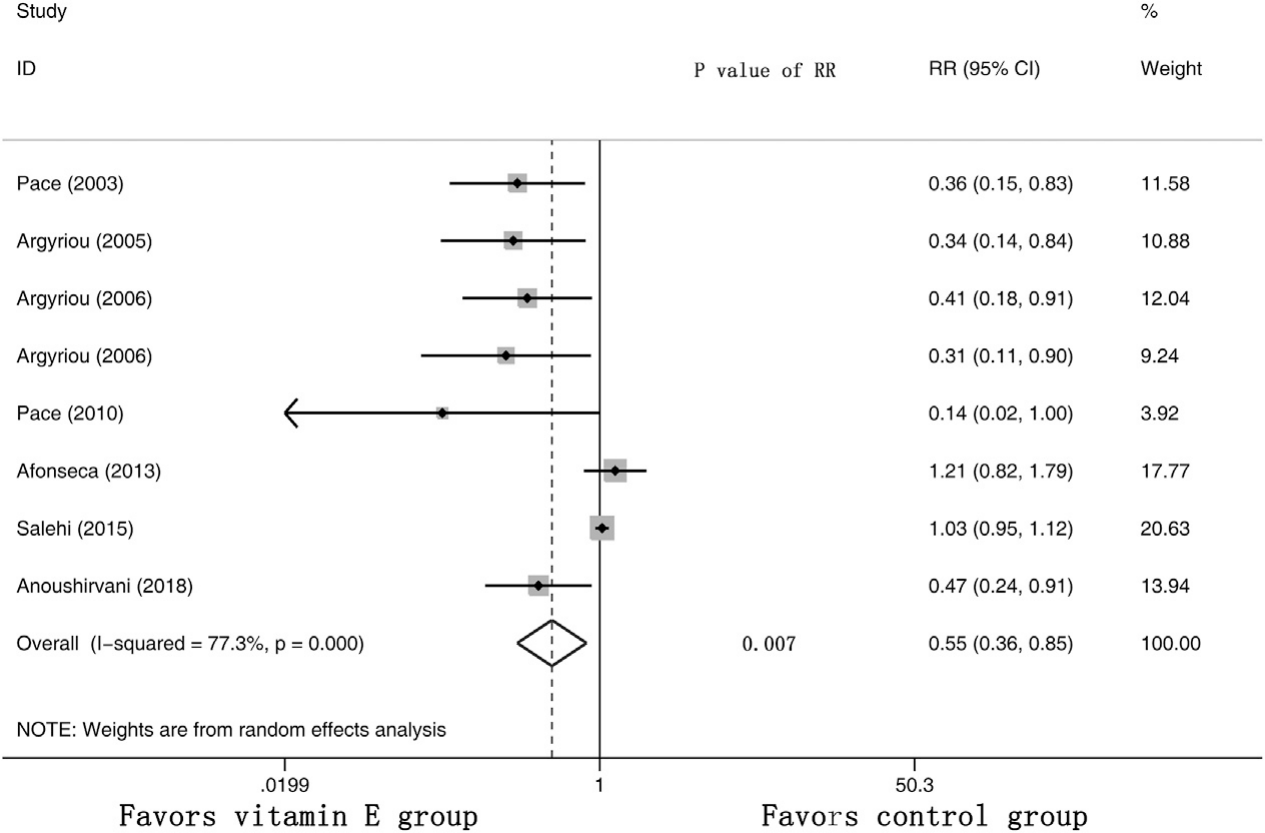
Forest plot of the incidence of all-grade CIPN in VE and control groups. Summary of the incidence of all-grade CIPN RR between VE and control groups were calculated using the random effect model. VE, vitamin E; CIPN, chemotherapy-induced peripheral neuropathy; RR, risk ratio; P, *p* value of the Q test for heterogeneity.

**FIGURE 4:**
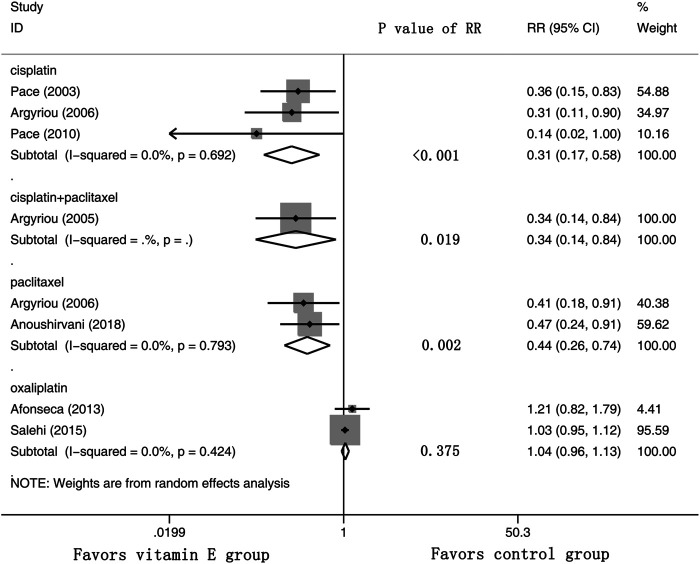
Forest plot of the subgroup analysis of incidence of all-grade CIPN in VE and control groups. Summary of the subgroup analysis of incidence of all-grade CIPN RR between VE and control groups were calculated using the random effect model. VE, vitamin E; CIPN, chemotherapy-induced peripheral neuropathy; RR, risk ratio; P, *p* value of the Q test for heterogeneity.

**FIGURE 5:**
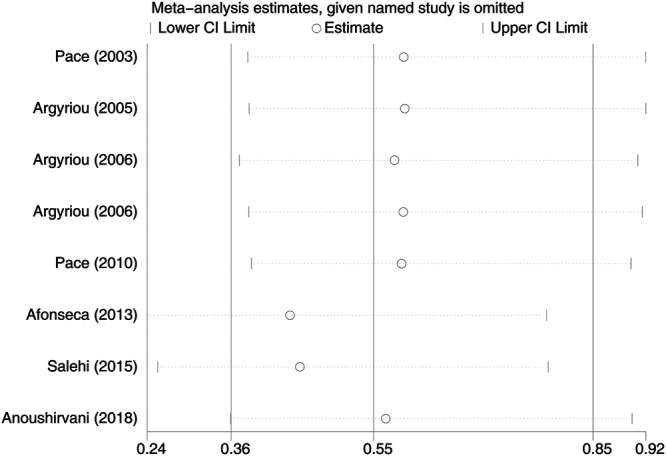
Sensitivity analysis of the incidence of all-grade CIPN in VE and control groups. VE, vitamin E; CIPN, chemotherapy-induced peripheral neuropathy.

Eight RCTs were evaluated for the effects of vitamin E on the incidence of all-grade CIPN (the raw data were listed in Supplementary Table S2). The results showed that vitamin E significantly reduced the incidence of all-grade CIPN in cancer patients (overall RR = 0.55, 95% CI: 0.36, 0.85, I^2^ = 77.3%, *p* = 0.007). The pooled effects on the incidence of all-grade CIPN are shown in Figure 3. I^2^ was greater than 50%, which demonstrates significant heterogeneity in this result. Therefore, a subgroup analysis was performed to identify the sources of heterogeneity. We analyzed the dose of vitamin E, the different regions of the patients, whether the trials were double-blind studies or not, and the different chemotherapy regimens were analyzed to find the sources of the heterogeneity (shown in Table 3 and Figure 4). The results suggested that the different chemotherapy regimens may be one of the sources of heterogeneity (shown in Figure 4). We also performed sensitivity analysis, which showed that the results on the incidence of all-grade CIPN were robust (shown in Figure 5). However, the results of the subgroup analysis which included only double-blind RCTs, suggested that vitamin E did not significantly reduce the incidence of all-grade CIPN (overall RR = 0.52, 95% CI: 0.07, 4.06, I^2^ = 77.5%, *p* = 0.531).

**TABLE 3:** Subgroup analysis of the incidence of all-grade CIPN.

Subgroup analysis	N	RR (95% CI)	*p* Values	Test for heterogeneity		
				Chi^2^	P_h_	I^2^
Dosage						
300 mg/d	1	0.36 (0.16, 0.83)	0.017	—	—	—
600 mg/d	4	0.40 (0.26, 0.60)	<0.001	0.54	0.909	0%
400 mg/d	3	1.03 (0.72, 1.46)	0.885	4.61	0.100	56.6%
Region						
Europe	5	0.34 (0.22, 0.53)	<0.001	1.00	0.910	0%
America	1	1.21 (0.82, 1.79)	0.333	—	—	—
Asian	2	0.74 (0.35, 1.60)	0.449	5.40	0.020	81.5%
Blinding						
Open-label	6	0.48 (0.27, 0.85)	0.012	26.03	<0.001	80.8%
Double-blind	2	0.52 (0.07, 4.06)	0.531	4.45	0.035	77.5%

CIPN, chemotherapy-induced peripheral neuropathy; N, number of trials; RR, risk ratio; Ph, *p* value of heterogeneity.

**FIGURE 6:**
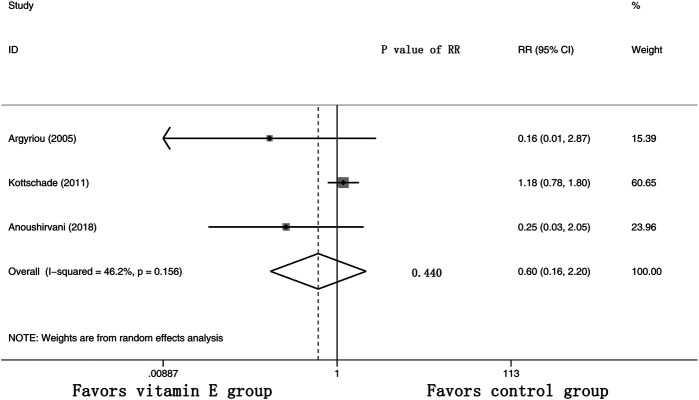
Forest plot of the incidence of severe CIPN in VE and control groups. Summary of the incidence of severe CIPN RR between VE and control groups were calculated using the random effect model. VE, vitamin E; CIPN, chemotherapy-induced peripheral neuropathy; RR, risk ratio; P, *p* value of the Q test for heterogeneity.

Furthermore, three RCTs were analyzed for the effects of vitamin E on the incidence of severe CIPN (the raw data were listed in Supplementary Table S3). The results showed that there was no significant difference in the incidence of severe CIPN between these two arms (overall RR = 0.60, 95% CI: 0.16, 2.20, I^2^ = 46.2%, *p* = 0.440). The pooled effects on the incidence of severe CIPN are shown in Figure 6.

### Evaluation of Total Neuropathy Scores

**FIGURE 7:**
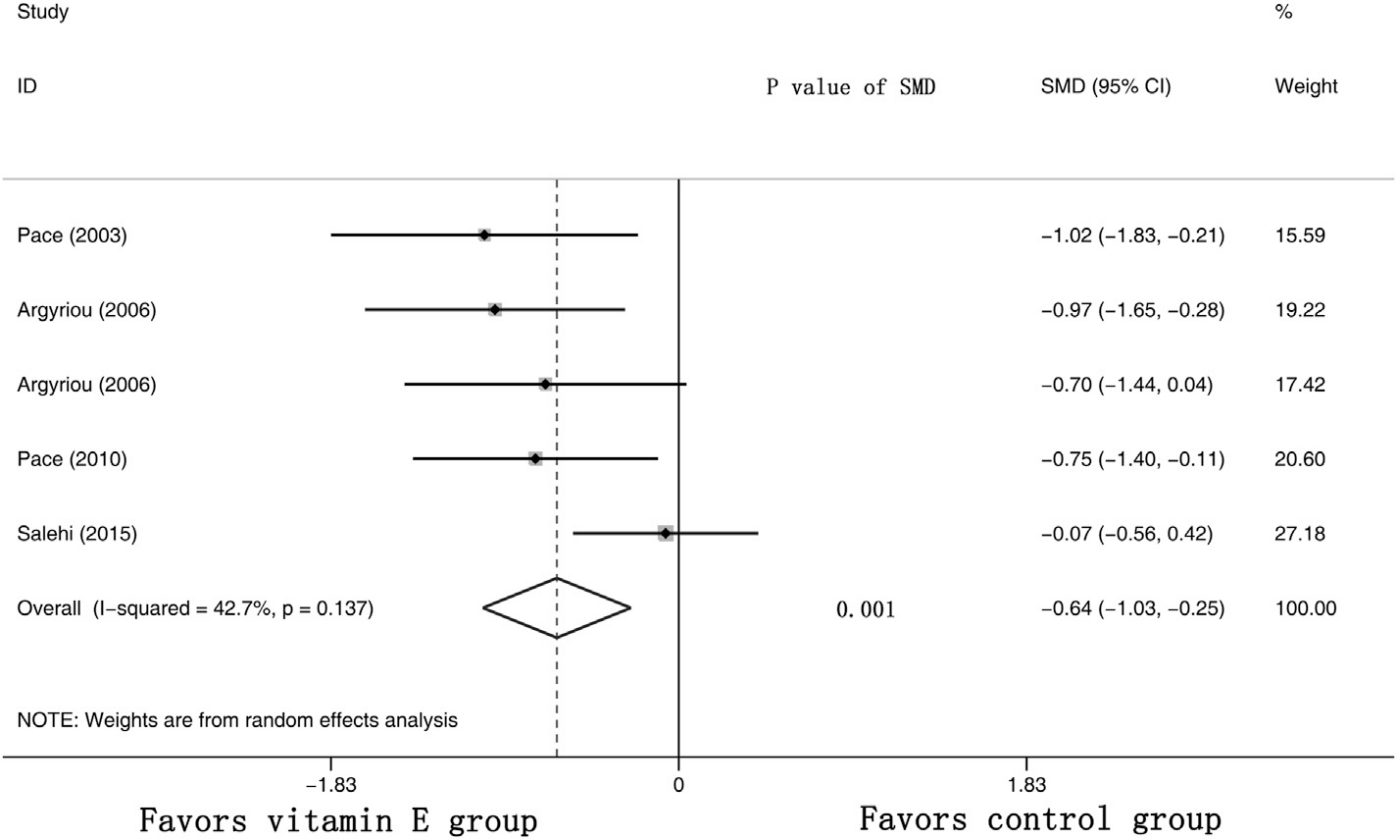
Forest plot of the total neuropathy scores in VE and control groups. Summary of the incidence of the total neuropathy scores SMD between VE and control groups were calculated using the random effect model. VE, vitamin E; CIPN, chemotherapy-induced peripheral neuropathy; SMD, standard mean differences; P, *p* value of the Q test for heterogeneity.

Five RCTs were assessed for the effects of vitamin E on the TNS (the raw data were listed in Supplementary Table S4). The results suggested that the TNS of the cancer patients was significantly lower in the vitamin E group than in the control group, with a pooled SMD of −0.64 (95% CI: −1.03, -0.25, I^2^ = 42.7%, *p* = 0.001) (shown in Figure 7).

### Publication Bias

**FIGURE 8:**
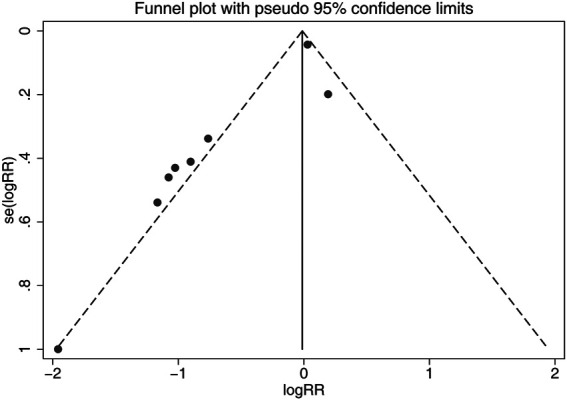
Funnel plots of differences of the incidence of all-grade CIPN in VE and control groups. VE, vitamin E; CIPN, chemotherapy-induced peripheral neuropathy.

Although the shapes of the funnel plots on the incidence of all-grade CIPN displayed partial asymmetry among the included studies, the results of the Begg’s test indicated no significant publication bias among these studies (*p* = 0.536). The funnel plots of the incidence of all-grade CIPN are shown in Figure 8.

## Discussion

Meta-analysis is considered to be an effective and widely used tool that can draw more meaningful conclusions than individual clinical studies by pooling results from different studies with specific statistical methods (Roberts et al., 2016; Lin and Chu, 2018). In this study, a total of 9 published RCTs were enrolled, which included 486 cancer patients. The pooled results demonstrated that vitamin E significantly reduced the incidence of all-grade CIPN (*p* = 0.007) and TNS (*p* = 0.001) in cancer patients. The results of this study were consistent with most of the RCTs included, indicating a beneficial effect of vitamin E on the incidence of CIPN. This may be explained by the fact that free radical damage caused by chemotherapy plays an important role in the production of CIPN (Bove et al., 2001; Velasco et al., 2016; Ahmad Bainmahfouz et al., 2021), and supplementation with vitamin E has revealed a potential role in preventing neuropathy in clinical settings (Rajanandh et al., 2014; Ng et al., 2020).

In this study, the results showed significant heterogeneity in the incidence of all-grade CIPN (I^2^ = 77.3%). Potential factors contributing to heterogeneity might include the dose of vitamin E, the different regions of the patients, whether the trials were double-blind studies or not, the different chemotherapy regimens, and other relevant factors. The results of the subgroup analysis suggested that the different chemotherapy regimens may be one of the sources of heterogeneity. We also performed sensitivity analysis, which showed that the results on the incidence of all-grade CIPN were robust. However, the results of the subgroup analysis, which included only double-blind RCTs, suggested that vitamin E did not significantly reduce the incidence of all-grade CIPN. This suggests that more high-quality double-blind RCTs are needed to verify this conclusion. The funnel plot of the incidence of all-grade CIPN showed some asymmetry, which suggests a possible publication bias. However, the results of the Begg’s test suggested that publication bias, if present, was theoretically negligible.

In comparison with the results of published meta-analyses or systematic reviews assessing the preventive effects of vitamin E on CIPN (Eum et al., 2013; Albers et al., 2014; Brami et al., 2016; Huang et al., 2016), this meta-analysis enrolled more RCTs. Although the results of the published meta-analysis were controversial, our findings provided further evidence that vitamin E has a beneficial effect on the incidence and symptoms of CIPN. However, most of the RCTs included in this meta-analysis were open-label, and CIPN assessment was subjective as mentioned in the introduction section. This suggests that routine prophylactic use of vitamin E is still not recommended, and more high-quality double-blind RCTs need to be conducted. There were only two RCTs comparing the incidence of adverse events between vitamin E and control groups; therefore, the number of RCTs was too small to perform a meta-analysis. However, the results of these two RCTs suggested that vitamin E for the prevention of CIPN did not significantly increase the incidence of adverse events (*p* > 0.05) (Argyriou et al., 2006b; Afonseca et al., 2013).

The results of this study may provide researchers and clinical practitioners with updated evidence regarding CIPN prevention in their practice fields. More large-sample double-blind RCTs on the use of vitamin E for the prevention of CIPN are still needed in the future, and investigators should use a rigorous methodological design to reduce the impact of potential bias on the results.

## Conclusion

Our results suggests that vitamin E has a beneficial effect on the incidence and symptoms of CIPN. However, the inclusion of open-label RCTs and subjective CIPN assessment suggests that routine prophylactic use of vitamin E is still not recommended. In addition, more high-quality double-blind RCTs are needed to validate the effects of vitamin E in prevention of CIPN.
