# Supplementary material for: Vitamin E for the Prevention of Chemotherapy-Induced Peripheral Neuropathy: A meta-Analysis
Source: Front Pharmacol. 2021 May 13;12:684550. doi: 10.3389/fphar.2021.684550 (PMC8155355; doi:10.3389/fphar.2021.684550)
Supplement: Supplementary file 2 [file Table2.docx]

**TABLE S2** Raw data of the incidence of all-grade CIPN in VE and control groups.

| **References** | **Studies** | **Total number of patients**  **(VE group)** | **Number of patients with all-grade CIPN**  **(VE group)** | **Total number of patients**  **(Control group)** | **Number of patients with all-grade CIPN**  **(VE group)** |
| --- | --- | --- | --- | --- | --- |
| Pace et al. (2003) | Pace 2003 | 13 | 4 | 14 | 12 |
| Argyriou et al. (2005) | Argyriou 2005 | 16 | 4 | 15 | 11 |
| Argyriou et al. (2006b) | Argyriou 2006 | 18 | 5 | 19 | 13 |
| Argyriou et al. (2006a) | Argyriou 2006 | 14 | 3 | 16 | 11 |
| Pace et al. (2010) | Pace 2010 | 17 | 1 | 24 | 10 |
| Afonseca et al. (2013) | Afonseca 2013 | 18 | 15 | 16 | 11 |
| Salehi et al. (2015) | Salehi 2015 | 32 | 32 | 33 | 32 |
| Anoushirvani et al. (2018) | Anoushirvani 2018 | 21 | 7 | 21 | 15 |

VE, vitamin E; CIPN, chemotherapy-induced peripheral neuropathy.
