# Supplementary material for: Vitamin E for the Prevention of Chemotherapy-Induced Peripheral Neuropathy: A meta-Analysis
Source: Front Pharmacol. 2021 May 13;12:684550. doi: 10.3389/fphar.2021.684550 (PMC8155355; doi:10.3389/fphar.2021.684550)
Supplement: Supplementary file 3 [file Table3.docx]

**TABLE S3** Raw data of the incidence of severe CIPN in VE and control groups.

| **References** | **Studies** | **Total number of patients**  **(VE group)** | **Number of patients with severe CIPN**  **(VE group)** | **Total number of patients**  **(Control group)** | **Number of patients with severe CIPN**  **(VE group)** |
| --- | --- | --- | --- | --- | --- |
| Argyriou et al. (2005) | Argyriou 2005 | 16 | 0 | 15 | 3 |
| Kottschade et al. (2011) | Kottschade 2011 | 96 | 33 | 93 | 27 |
| Anoushirvani et al. (2018) | Anoushirvani 2018 | 21 | 1 | 21 | 4 |

VE, vitamin E; CIPN, chemotherapy-induced peripheral neuropathy.
