# Supplementary material for: Vitamin E for the Prevention of Chemotherapy-Induced Peripheral Neuropathy: A meta-Analysis
Source: Front Pharmacol. 2021 May 13;12:684550. doi: 10.3389/fphar.2021.684550 (PMC8155355; doi:10.3389/fphar.2021.684550)
Supplement: Supplementary file 4 [file Table4.docx]

**TABLE S4** Raw data of the total neuropathy scores of patients in VE and control groups.

| **References** | **Studies** | **Total number of patients**  **(VE group)** | **TNS of patients**  **(Mean ± SD, VE group)** | **Total number of patients**  **(Control group)** | **TNS of patients**  **(Mean ± SD, Control group)** |
| --- | --- | --- | --- | --- | --- |
| Pace et al. (2003) | Pace 2003 | 13 | 2.1±2.1 | 14 | 4.7±2.9 |
| Argyriou et al. (2006b) | Argyriou 2006 | 18 | 2.2±5.1 | 19 | 11.0±11.6 |
| Argyriou et al. (2006a) | Argyriou 2006 | 14 | 5.0±1.3 | 16 | 10.5±10.6 |
| Pace et al. (2010) | Pace 2010 | 17 | 1.4±1.5 | 24 | 4.1±4.5 |
| Salehi et al. (2015) | Salehi 2015 | 32 | 6.4±2.8 | 33 | 6.6±2.9 |

VE, vitamin E; TNS, total neuropathy scores.
